# A systematic review on fake news research through the lens of news creation and consumption: Research efforts, challenges, and future directions

**DOI:** 10.1371/journal.pone.0260080

**Published:** 2021-12-09

**Authors:** Bogoan Kim, Aiping Xiong, Dongwon Lee, Kyungsik Han

**Affiliations:** 1 School of Intelligence Computing, Hanyang University, Seoul, Republic of Korea; 2 College of Information Sciences and Technology, Pennsylvania State University, State College, PA, United States of America; Universita degli Studi della Campania Luigi Vanvitelli, ITALY

## Abstract

**Background:**

Although fake news creation and consumption are mutually related and can be changed to one another, our review indicates that a significant amount of research has primarily focused on news creation. To mitigate this research gap, we present a comprehensive survey of fake news research, conducted in the fields of computer and social sciences, through the lens of *news creation* and *consumption* with internal and external factors.

**Methods:**

We collect 2,277 fake news-related literature searching six primary publishers (ACM, IEEE, arXiv, APA, ELSEVIER, and Wiley) from July to September 2020. These articles are screened according to specific inclusion criteria (see Fig 1). Eligible literature are categorized, and temporal trends of fake news research are examined.

**Results:**

As a way to acquire more comprehensive understandings of fake news and identify effective countermeasures, our review suggests (1) developing a computational model that considers the characteristics of news consumption environments leveraging insights from social science, (2) understanding the diversity of news consumers through mental models, and (3) increasing consumers’ awareness of the characteristics and impacts of fake news through the support of transparent information access and education.

**Conclusion:**

We discuss the importance and direction of supporting one’s “digital media literacy” in various news generation and consumption environments through the convergence of computational and social science research.

## 1 Introduction

The spread of fake news not only deceives the public, but also affects society, politics, the economy and culture. For instance, Buzzfeed (https://www.buzzfeed.com/) compared and analyzed participation in 20 real news and 20 fake news articles (e.g., likes, comments, share activities) that spread the most on Facebook during the last three months of the 2016 US Presidential Election. According to the results, the participation rate of fake news (8.7 million) was higher than that of mainstream news (7.3 million), and 17 of the 20 fake news played an advantageous role in winning the election [1]. Pakistan’s ministry of Defense posted a tweet fiercely condemning Israel after coming to believe that Israel had threatened Pakistan with nuclear weapons, which was later found to be false [2]. Recently, the spread of the absurd rumor that COVID-19 propagates through 5G base stations in the UK caused many people to become upset and resulted in a base station being set on fire [3].

Such fake news phenomenon has been rapidly evolving with the emergence of social media [4, 5]. Fake news can be quickly shared by friends, followers, or even strangers within only a few seconds. Repeating a series of these processes could lead the public to form the wrong collective intelligence [6]. This could further develop into diverse social problems (i.e., setting a base station on fire because of rumors). In addition, some people believe and propagate fake news due to their personal norms, regardless of the factuality of the content [7]. Research in social science has suggested that cognitive bias (e.g., confirmation bias, bandwagon effect, and choice-supportive bias) [8] is one of the most pivotal factors in making irrational decisions in terms of the both creation and consumption of fake news [9, 10]. Cognitive bias greatly contributes to the formation and enhancement of the echo chamber [11], meaning that news consumers share and consume information only in the direction of strengthening their beliefs [12].

Research using computational techniques (e.g., machine or deep learning) has been actively conducted for the past decade to investigate the current state of fake news and detect it effectively [13]. In particular, research into text-based feature selection and the development of detection models has been very actively and extensively conducted [14–17]. Research has been also active in the collection of fake news datasets [18, 19] and fact-checking methodologies for model development [20–22]. Recently, Deepfake, which can manipulate images or videos through deep learning technology, has been used to create fake news images or videos, significantly increasing social concerns [23], and a growing body of research is being conducted to find ways of mitigating such concerns [24–26]. In addition, some research on system development (i.e., a game to increase awareness of the negative aspects of fake news) has been conducted to educate the public to avoid and prevent them from the situation where they could fall into the echo chamber, misunderstandings, wrong decision-making, blind belief, and propagating fake news [27–29].

**Fig 1:**
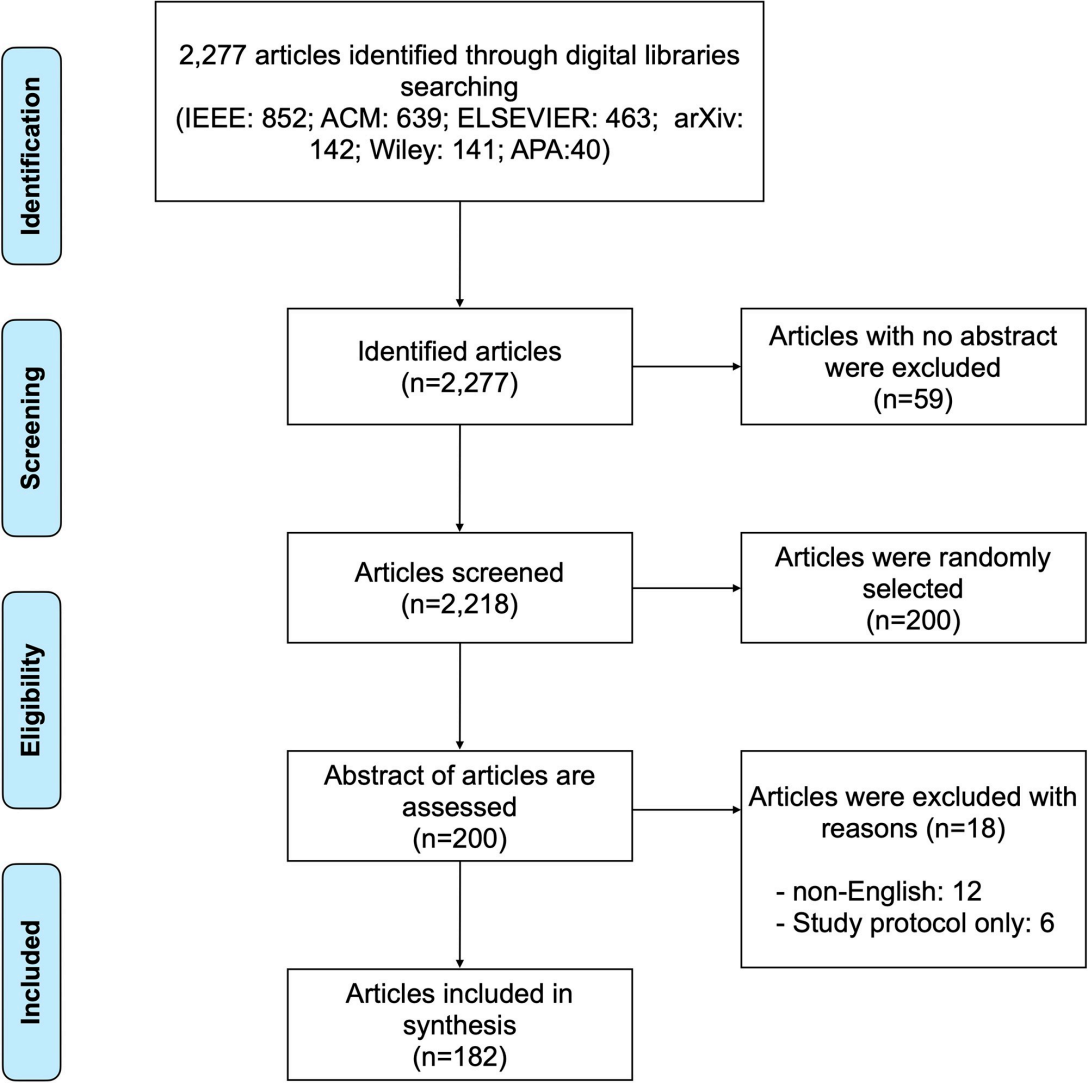
PRISMA flow chart of literature search for fake news from 2010 to 2020.

**Fig 2:**
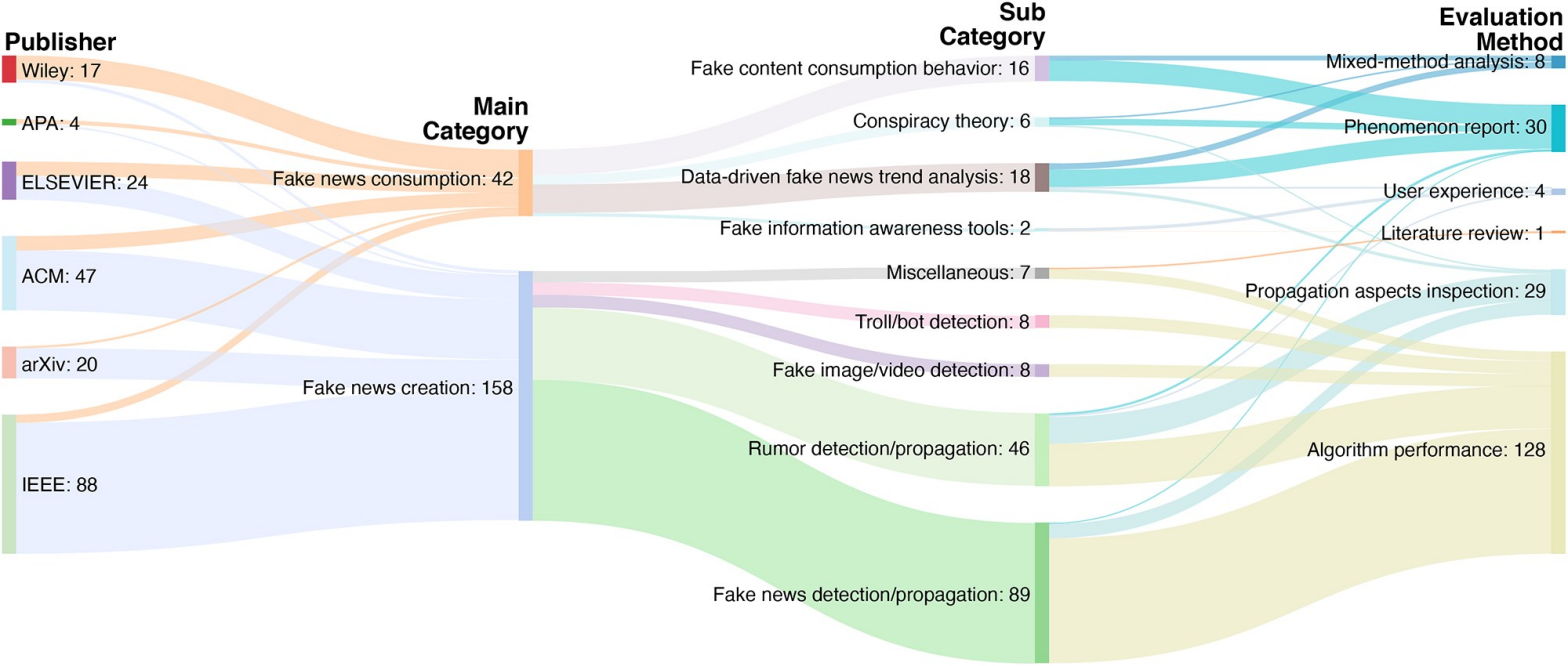
A visualization of 182 fake news-related papers. The papers were published in IEEE, ACM, ELSEVIER, arXiv, Wiley, APA from 2010 to 2020 classified by publisher, main category, sub category, and evaluation method (left to right).

While the creation and consumption of fake news are clearly different behaviors, due to the characteristics of the online environment (e.g., information can be easily created, shared, and consumed by anyone at anytime from anywhere), the boundaries between fake news creators and consumers have started to become blurred. Depending on the situation, people can quickly change their roles from fake news consumers to creators, or vice versa (with or without their intention). Furthermore, news creation and consumption are the most fundamental aspects that form the relationship between news and people. However, a significant amount of fake news research has positioned in news creation while considerably less research focus has been placed in news consumption (see Figs 1 & 2). This suggests that we must consider fake news *as a comprehensive aspect of news consumption and creation*.

**Fig 3:**
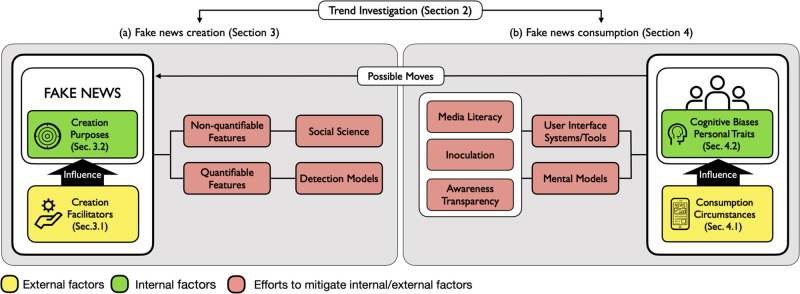
An overview of the survey on fake news research in this paper. We investigate fake news research trend (Section 2), and examine fake news creation and consumption through the lenses of external and internal factors. We also investigate research efforts to mitigate external factors of fake news creation and consumption: (a) indicates fake news creation (Section 3), and (b) indicates fake news consumption (Section 4). “Possible moves” indicates that news consumers “possibly” create/propagate fake news without being aware of any negative impact.

In this paper, we looked into fake news research through the lens of news creation and consumption (Fig 3). Our survey results offer different yet salient insights on fake news research compared with other survey papers (e.g., [13, 30, 31]), which primarily focus on fake news creation. The main contributions of our survey are as follows:

- We investigate trends in fake news research from 2010 to 2020 and confirm a need for applying a comprehensive perspective to fake news phenomenon.
- We present fake news research through the lens of news creation and consumption with external and internal factors.
- We examine key findings with a mental model approach, which highlights individuals’ differences in information understandings, expectations, or consumption.
- We summarize our review and discuss complementary roles of computer and social sciences and potential future directions for fake news research.

## 2 Fake news definition and trends

There is still no definition of fake news that can encompass false news and various types of disinformation (e.g., satire, fabricated content) and can reach a social consensus [30]. The definition continues to change over time and may vary depending on the research focus. Some research has defined fake news as false news based on the intention and factuality of the information [4, 15, 32–36]. For example, Allcott and Gentzkow [4] defined fake news as “news articles that are intentionally and verifiably false and could mislead readers.” On the other hand, other studies have defined it as “a news article or message published and propagated through media, carrying false information regardless of the means and motives behind it” [13, 37–43]. Given this definition, fake news refers to false information that causes an individual to be deceived or doubt the truth, and fake news can only be useful if it actually deceives or confuses consumers. Zhou and Zafarani [31] proposed a *broad* definition (“Fake news is false news.”) that encompasses false online content and a *narrow* definition (“Fake news is intentionally and verifiably false news published by a news outlet.”). The narrow definition is valid from the fake news creation perspective. However, given that fake news creators and consumers are now interchangeable (e.g., news consumers also play a role of gatekeeper for fake news propagation), it has become important to understand and investigate the fake news through consumption perspectives. Thus, in this paper, we use the broad definition of fake news.

Our research motivation for considering news creation and consumption in fake news research was based on the trend analysis. We collected 2,277 fake news-related literature using four keywords (i.e., fake news, false information, misinformation, rumor) to identify longitudinal trends of fake news research from 2010 to 2020. The data collection was conducted from July to September 2020. The criteria of data collection was whether any of these keywords exists in the title or abstract. To reflect diverse research backgrounds/domains, we considered six primary publishers (ACM, IEEE, arXiv, APA, ELSEVIER, and Wiley). The number of papers collected for each publisher is as follows: 852 IEEE (37%), 639 ACM (28%), 463 ELSEVIER (20%), 142 arXiv (7%), 141 Wiley (6%), 40 APA (2%). We excluded 59 papers that did not have the abstract and used 2,218 papers for the analysis. We then randomly chose 200 papers, and two coders conducted manual inspection and categorization. The inter-coder reliability was verified by the Cohen’s Kappa measurement. The scores for each main/sub-category were higher than 0.72 (min: 0.72, max: 0.95, avg: 0.85), indicating that the inter-coder reliability lies between “substantial” to “perfect” [44]. Through the coding procedure, we excluded non-English studies (n = 12) and reports on study protocol only (n = 6), and 182 papers were included in synthesis. The PRISMA flow chart depicts the number of articles identified, included, and excluded (see Fig 1).

**Fig 4:**
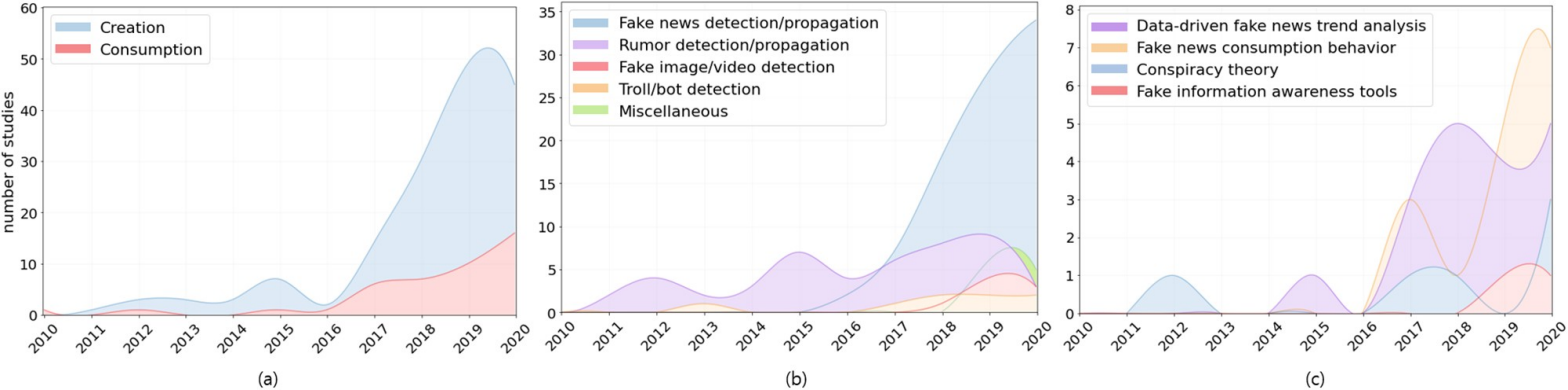
Fake news research trends of the past decade (2010–2020). We collected 2,277 fake news related-papers and randomly chose and categorized 200 papers. Each marker indicates the number of fake news studies per type published in a given year. Fig 4(a) shows a research trend of news creation and consumption (main category). Fig 4(b) and 4(c) show a trend of the sub-categories of news creation and consumption. In Fig 4(b), “Miscellaneous” includes studies on stance/propaganda detection and a survey paper. In Fig 4(c), “Data-driven fake news trend analysis” mainly covers the studies reporting the influence of fake news that spread around specific political/social events (e.g., fake news in Presidential Election 2016, Rumor in Weibo after 2015 Tianjin explosions). “Conspiracy theory” refers to an unverified rumor that was passed on to the public.

The papers were categorized into two main categories: (1) creation (studies with efforts to detect fake news or mitigate spread of fake news) and (2) consumption (studies that reported the social impacts of fake news on individuals or societies and how to appropriately handle fake news). Each main category was then classified into sub-categories. Fig 4 shows the frequency of the entire literature by year and the overall trend of fake news research. It appears that the consumption perspective of fake news still has not received sufficient attention compared with the creation perspective (Fig 4(a)). Fake news studies have exploded since the 2016 US Presidential Election, and the trend of increase in fake news research continues. In the creation category, the majority of papers (135 out of 158; 85%) were related to the false information (e.g., fake news, rumor, clickbait, spam) detection model (Fig 4(b)). On the other hand, in the consumption category, much research pertains to data-driven fake news trend analysis (18 out of 42; 43%) or fake content consumption behavior (16 out of 42; 38%), including studies for media literacy education or echo chamber awareness (Fig 4(c)).

## 3 Fake news creation

Fake news is no longer merely propaganda spread by inflammatory politicians; it is also made for financial benefit or personal enjoyment [45]. With the development of social media platforms people often create completely false information for reasons beyond satire. Further, there is a vicious cycle of this false information being abused by politicians and agitators.

Fake news creators are indiscriminately producing fake news while considering the behavioral and psychological characteristics of today’s news consumers [46]. For instance, the sleeper effect [47] refers to a phenomenon in which the persuasion effect increases over time, even though the pedigree of information shows low reliability. In other words, after a long period of time, memories of the pedigree become poor and only the content tends to be remembered regardless of the reliability of the pedigree. Through this process, less reliable information becomes more persuasive over time. Fake news creators have effectively created and propagated fake news by targeting the public’s preference for news consumption through peripheral processing routes [35, 48].

Peripheral routes are based on the elaboration likelihood model (ELM) [49], one of the representative psychological theories that handles persuasive messages. According to the ELM, the path of persuasive message processing can be divided into the central and the peripheral routes depending on the level of involvement. On one hand, if the message recipient puts a great deal of cognitive effort into processing, the central path is chosen. On the other hand, if the process of the message is limited due to personal characteristics or distractions, the peripheral route is chosen. Through a peripheral route, a decision is made based on other secondary cues (e.g., speakers, comments) rather than the logic or strength of the argument.

Wang et al. [50] demonstrated that most of the links shared or mentioned in social media have never even been clicked. This implies that many people perceive and process information in only fragmentary way, such as via news headlines and the people sharing news, rather than considering the logical flow of news content.

In this section, we closely examined each of the external and internal factors affecting fake news creation, as well as the research efforts carried out to mitigate the negative results based on the fake news creation perspective.

### 3.1 External factors: Fake news creation facilitators

**Fig 5:**
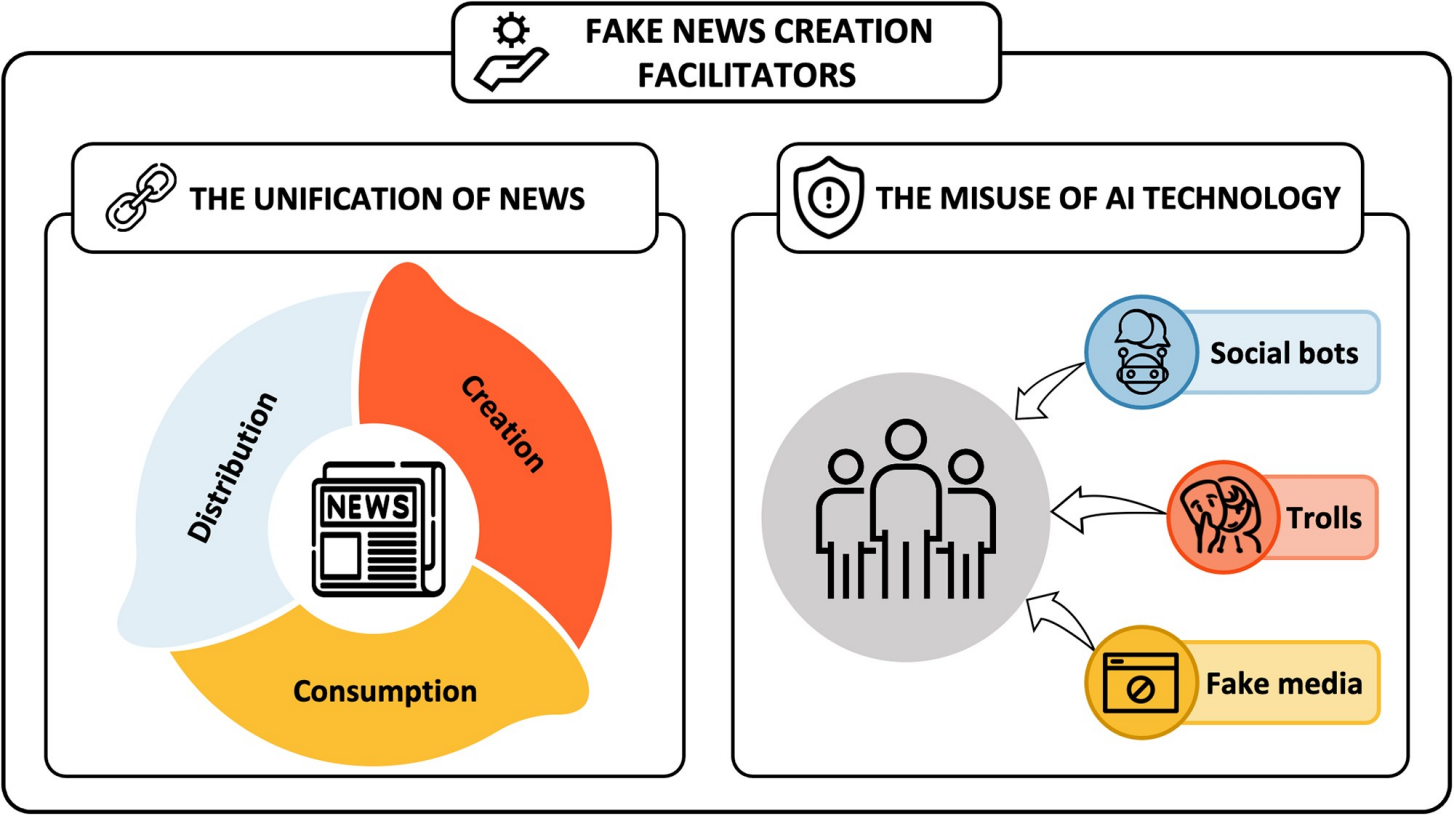
External factors: Fake news creation facilitators. We identify two external factors—The unification of news and the misuse of AI technology—That facilitate fake news creation.

We identified two external factors that facilitate fake news creation and propagation: *(1) the unification of news creation, consumption, and distribution, (2) the misuse of AI technology, and (3) the use of social media as a news platform* (see Fig 5).

#### 3.1.1 The unification of news creation, consumption, and distribution

The public’s perception of news and the major media of news consumption has gradually changed. The public no longer passively consumes news exclusively through traditional news organizations with specific formats (e.g., the inverted pyramid style, verified sources) nor view those news simply as a medium for information acquisition. The public’s active news consumption behaviors began in earnest with the advent of citizen journalism by implementing journalistic behavior based on citizen participation [51] and became commonplace with the emergence of social media. As a result, the public began to prefer interactive media, in which new information could be acquired, their opinions can be offered, and they can discuss the news with other news consumers. This environment has motivated the public to make content about their beliefs and deliver the content to many people as “news.” For example, a recent police crackdown video posted in social media quickly spread around the world that influenced protesters and civic movements. Then, it was reported later by the mainstream media [52].

The boundaries between professional journalists and amateurs, as well as between news consumers and creators, are disappearing. This has led to a potential increase in deceptive communications, making news consumers suspicious and misinterpreted the reality. Online platforms (e.g., YouTube, Facebook) that allow users to freely produce and distribute content have been growing significantly. As a result, fake news content can be used to attract secondary income (e.g., multinational enterprises’ advertising fees), which contributes to accelerating fake news creation and propagation. An environment in which the public can only consume news that suits their preferences and personal cognitive biases has made it much easier for fake news creators to achieve their specific purposes (e.g., supporting a certain political party or a candidate they favor).

#### 3.1.2 The misuse of AI technology

The development of AI technology has made it easier to develop and utilize tools for creating fake news, and many studies have confirmed the impact of these technologies—*(1) social bots, (2) trolls, and (3) fake media*—on social networks and democracy over the past decade.

*3.1.2.1 Social bots*. Shao et al. [53] analyzed the pattern of fake news spread and confirmed that social bots play a significant role in fake news propagation and social bot-based automated accounts were largely affected by the initial stage of spreading fake news. In general, it is uneasy for the public to determine whether such accounts are people or bots. In addition, social bots are not illegal tools and many companies legally purchase them as a part of marketing, thus it is not easy to curb the use of social bots systematically.

*3.1.2.2 Trolls*. The term “trolls” refers to people who deliberately cause conflict or division by uploading inflammatory, provocative content or unrelated posts to online communities. They work with the aim of stimulating people’s feelings or beliefs and hindering mature discussions. For example, the Russian troll army has been active in social media to advance its political agenda and cause social turmoil in the US [54]. Zannettou et al. [55] confirmed how effectively the Russian troll army has been spreading fake news URLs on Twitter and its significant impact on making other Twitter users believe misleading information.

*3.1.2.3 Fake media*. It is now possible to manipulate or reproduce content in 2D or even 3D through AI technology. In particular, the advent of fake news using Deepfake technology (combining various images on an original video and generating a different video) has raised another major social concern that had not been imagined before. Due to the popularity of image or video sharing on social media, such media types have become the dominant form of news consumption, and the Deepfake technology itself is becoming more advanced and applied to images and videos in a variety of domains. We witnessed a video clip of former US President Barack Obama criticizing Donald Trump, which was manipulated by the US online media company BuzzFeed to highlight the influence and danger of Deepfake, causing substantial social confusion [56].

### 3.2 Internal factors: Fake news creation purposes

We identified three main purposes for fake news creation—*(1) ideological purposes, (2) monetary purposes, and (3) fear/panic reduction*.

#### 3.2.1 Ideological purpose

Fake news has been created and propagated for political purposes by individuals or groups that positively affect the parties or candidates they support or undermine those who are not on the same side. Fake news with this political purpose has shown to negatively influence people and society. For instance, Russia created a fake Facebook account that caused many political disputes and enhanced polarization, affecting the 2016 US Presidential Election [57]. As polarization has intensified, there has also been a trend in the US that “unfriending” people who have different political tendencies [58]. This has led the public to decide whether to trust the news or not regardless of its factuality and has resulted in worsening in-group biases. During the Brexit campaign in the UK, many selective news articles were exposed on Facebook, and social bots and trolls were also confirmed as being involved in creating public opinions [59, 60].

#### 3.2.2 Monetary purpose

Financial benefit is another strong motivation for many fake news creators [34, 61]. Fake news websites usually reach the public through social media and make profits through posted advertisements. The majority of fake websites are focused on earning advertising revenue by spreading fake news that would attract readers’ attention, rather than political goals. For example, during the 2016 US Presidential Election in Macedonia, young people in their 10s and 20s used content from some extremely right-leaning blogs in the US to mass-produce fake news, earning huge advertising revenues [62]. This is also why fake news creators use provocative titles, such as clickbait headlines, to induce clicks and attempt to produce as many fake news articles as possible.

#### 3.2.3 Fear and panic reduction

In general, when epidemics become more common around the world, rumors of absurd and false medical tips spread rapidly in social media. When there is a lack of verified information, people feel great anxious and afraid and easily believe such tips, regardless of whether they are true [63, 64]. The term *infodemic*, which first appeared during the 2003 SARS pandemics, describes this phenomenon [65]. Regarding COVID-19, health authorities have recently announced that preventing the creation and propagation of fake news about the virus is as important as alleviating the contagious power of COVID-19 [66, 67]. The spread of fake news due to the absence of verified information has become more common regarding health-related social issues (e.g., infectious diseases), natural disasters, etc. For example, people with disorders affecting cognition (e.g., neurodegenerative disorder) are tend to easily believe unverified medical news [68–70]. Robledo and Jankovic [68] confirmed that many fake or exaggerated medical journals are misleading people with Parkinson’s disease by giving false hopes and unfounded fake articles. Another example is a rumor that climate activists set fire to raise awareness of climate change quickly spread as fake news [71], when a wildfire broke out in Australia in 2019. As a result, people became suspicious and tended to believe that the causes of climate change (e.g., global warming) may not be related to humans, despite scientific evidence and research data.

### 3.3 Fake news detection and prevention

The main purpose of fake news creation is to make people confused or deceived regardless of topic, social atmosphere, or timing. Due to this purpose, it appears that fake news tends to have similar frames and structural patterns. Many studies have attempted to mitigate the spread of fake news based on these identifiable patterns. In particular, research on developing computational models that detect fake information (text/images/videos), based on machine or deep learning techniques has been actively conducted, as summarized in Table 1. Other modeling studies include the credibility of weblogs [84, 85], communication quality [88], susceptibility level [90], and political stance [86, 87]. The table was intended to characterize a research scope and direction of the development of fake information creation (e.g., the features employed in each model development), not to present an exhaustive list.

**Table 1:** Examples of the prior research on fake information and visual media detection modeling.

Focus	Authors	Linguistics (Text)	Vision (Image)	Sentiment	Topic	User	Network	Data Source
Fake news	Cui et al. [16]	✓	✓	✓		✓	✓	Politifact & GossipCop
	arimi et al. [72]	✓				✓		LIAR [73]
	Pérez-Rosas et al. [74]	✓			✓			News websites in US
	Ruchansky et al. [15]	✓				✓		Twitter & Weibo (microblogging)
	Shu et al. [14]	✓	✓			✓	✓	Politifact, GossipCop
	Wang et al. [17]	✓	✓			✓		Twitter & Weibo
	Yang et al. [75]	✓	✓	✓	✓			News websites in US
Clickbait	Kumar et al. [76]	✓	✓					Twitter
	Yoon et al. [77]	✓						News articles in Korea
Fake review	Lu et al. [78]	✓				✓		Amazon
	Mukgerjee et al. [79]	✓				✓		Yelp
Spammer	Benevenuto et al. [80]	✓				✓		Twitter
	Lee et al. [81]					✓		My space, Twitter
Spam review	Li et al. [82]	✓		✓		✓		Epinions (general consumer review site)
Fraudster	Wang et al. [83]	✓				✓	✓	Tencent App Store
Information credibility	Castillo et al. [84]	✓		✓	✓	✓	✓	Twitter
	Jo et al. [85]	✓		✓		✓		Naver (microblogging)
Political stance	Che et al. [86]	✓						News websites in US
	Potthast et al. [87]	✓			✓			News websites in US
Communication quality	Han et al. [88]	✓		✓		✓		Twitter
Text credibility	Popat et al. [89]	✓						Snopes
Suscepitble level	Shen et al. [90]	✓				✓	✓	Twitter
Fake image	Gupta et al. [91]	✓	✓	✓		✓		Twitter
	He et al. [92]		✓					CelebFaces [93]
	Huh et al. [94]		✓					Flickr
	Stehouwer et al. [95]		✓					DFFD
	Tariq et al. [96]		✓					CelebA [97]
	Wang et al. [98]		✓					CelebA [97], FFHQ [99]
	Yang et al. [100]		✓					CelebA [97]
	Zhang et al. [101]		✓					MSCOCO
Deepfake	Amerini et al. [102]		✓					FaceForensics++ [25]
	Li & Lyu [103]		✓					UADFV [100], DeepfakeTIMIT [104]
Fake talker	Jeon et al. [105]		✓					VoxCeleb2 [106]
Face forensic	Songsri-in et al. [107]		✓					CelebA [97], FFHQ [99]
Visual reasoning	Ma et al. [108]	✓	✓					Bilibili (Chinese video sharing website)

#### 3.3.1 Fake text information detection

Research has considered many text-based features, such as structural (e.g., website URLs and headlines with all capital letters or exclamations) and linguistic information (e.g., grammar, spelling, and punctuation errors) about the news. Research has also considered the sentiments of news articles, the frequency of the words used, user information, and who left comments on the news articles, and social network information among users (who were connected based on activities of commenting, replying, liking or following) were used as key features for model development. These text-based models have been developed for not only fake news articles but also other types of fake information, such as clickbaits, fake reviews, spams, and spammers. Many of the models developed in this context performed a binary classification that distinguished between fake and non-fake articles, with the accuracy of such models ranging from 86% to 93%. Mainstream news articles were used to build most models, and some studies used articles on social media, such as Twitter [15, 17]. Some studies developed fake news detection models by extracting features from images, as well as text, in news articles [16, 17, 75].

#### 3.3.2 Fake visual media detection

The generative adversary network (GAN) is an unsupervised learning method that estimates the probability distribution of original data and allows an artificial neural network to produce similar distributions [109]. With the advancement of GAN, it has become possible to transform faces in images into those of others. However, photos of famous celebrities have been misused (e.g., being distorted into pornographic videos), increasing concerns about the possible misuse of such technology [110] (e.g., creating rumors about a certain political candidate). To mitigate this, research has been conducted to develop detection models for fake images. Most studies developed binary classification models (fake image or not), and the accuracy of fake image detection models was high, ranging from 81% to 97%. However, challenges still exist. Unlike fake news detection models that employ fact-checking websites or mainstream news as data verification or ground-truth, fake image detection models were developed using the same or slightly modified image datasets (e.g., CelebA [97], FFHQ [99]), asking for the collection and preparation of a large amount of highly diverse data.

## 4 Fake news consumption

### 4.1 External factors: Fake news consumption circumstances

The implicit social contract between civil society and the media has gradually disintegrated in modern society, and accordingly, citizens’ trust in the media began to decline [111]. In addition, the growing number of digital media platforms has changed people’s news consumption environment. This change has increased the diversity of news content and the autonomy of information creation and sharing. At the same time, however, it blurred the line between traditional mainstream media news and fake news in the Internet environment, contributing to polarization.

Here, we identified three external factors that have forced the public to encounter fake news: *(1) the decline of trust in the mainstream media, (2) a high-choice media environment, and (3) the use of social media as a news platform*.

#### 4.1.1 Fall of mainstream media trust

Misinformation and unverified or biased reports have gradually undermined the credibility of the mainstream media. According to the 2019 American mass media trust survey conducted by Gallup, only 13% of Americans said they trusted traditional mainstream media: newspapers or TV news [112]. The decline in traditional media trust is not only a problem for the US, but also a common concern in Europe and Asia [113–115].

#### 4.1.2 High-choice media environment

Over the past decade, news consumption channels have been radically diversified, and the mainstream has shifted from broadcasting and print media to mobile and social media environments. Despite the diversity of news consumption channels, personalized preferences and repetitive patterns have led people to be exposed to limited information and continue to consume such information increasingly [116]. This selective news consumption attitude has enhanced the polarization of the public in many multi-media environments [117]. In addition, the commercialization of digital platforms have created an environment in which cognitive bias can be easily strengthened. In other words, a digital platform based on recommended algorithms has the convenience of providing similar content continuously after a given type of content is consumed. As a result, it may be easy for users to fall into the echo chamber because they only access recommended content. A survey of 1,000 YouTube videos found that more than two-thirds of the videos contained content in favor of a particular candidate [118].

News consumption in social media does not simply mean the delivery of messages from creators to consumers. The multi-directionality of social media has blurred the boundaries between information creators and consumers. In other words, users are already interacting with one another in various fashions, and when a new interaction type emerges and is supported by the platform, users will display other types of new interactions, which will also influence ways of consuming news information.

#### 4.1.3 Use of social media as news platform

Here we focus on the most widely used social media platforms—YouTube, Facebook, and Twitter—where each has characteristics of encouraging limited news consumption.

First, YouTube is the most *unidirectional* of social media. Many YouTube creators tend to convey arguments in a strong, definitive tone through their videos, and these content characteristics make viewers judge the objectivity of the information via non-verbal elements (e.g., speaker, thumbnail, title, comments) rather than facts. Furthermore, many comments often support the content of the video, which may increase the chances of viewers accepting somewhat biased information. In addition, a YouTube video recommendation algorithm causes users who watch certain news to continuously be exposed to other news containing the same or similar information. This behavior and direction on the part of isolated content consumption could undermine the viewer’s media literacy, and is likely to create a screening effect that blocks the user’s eyes and ears.

Second, Facebook is somewhat *invisible* regarding the details of news articles because this platform ostensibly shows only the title, the number of likes, and the comments of the posts. Often, users have to click on the article and go to the URL to read the article. This structure and consumptive content orientation on the part of Facebook presents obstacles that prevent users from checking the details of their posts. As a result, users have become likely to make limited and biased judgments and perceive content through provocative headlines and comments.

Third, the largest feature of Twitter is *anonymity* because Twitter asks users to make their own pseudonyms [119]. Twitter has a limited number of letters to upload, and compared to other platforms, users can produce and spread indiscriminate information anonymously and do not know who is behind the anonymity [120, 121]. On the other hand, many accounts on Facebook operate under real names and generally share information with others who are friends or followers. Information creators are not held accountable for anonymous information.

### 4.2 Internal factors: Cognitive mechanism

**Fig 6:**
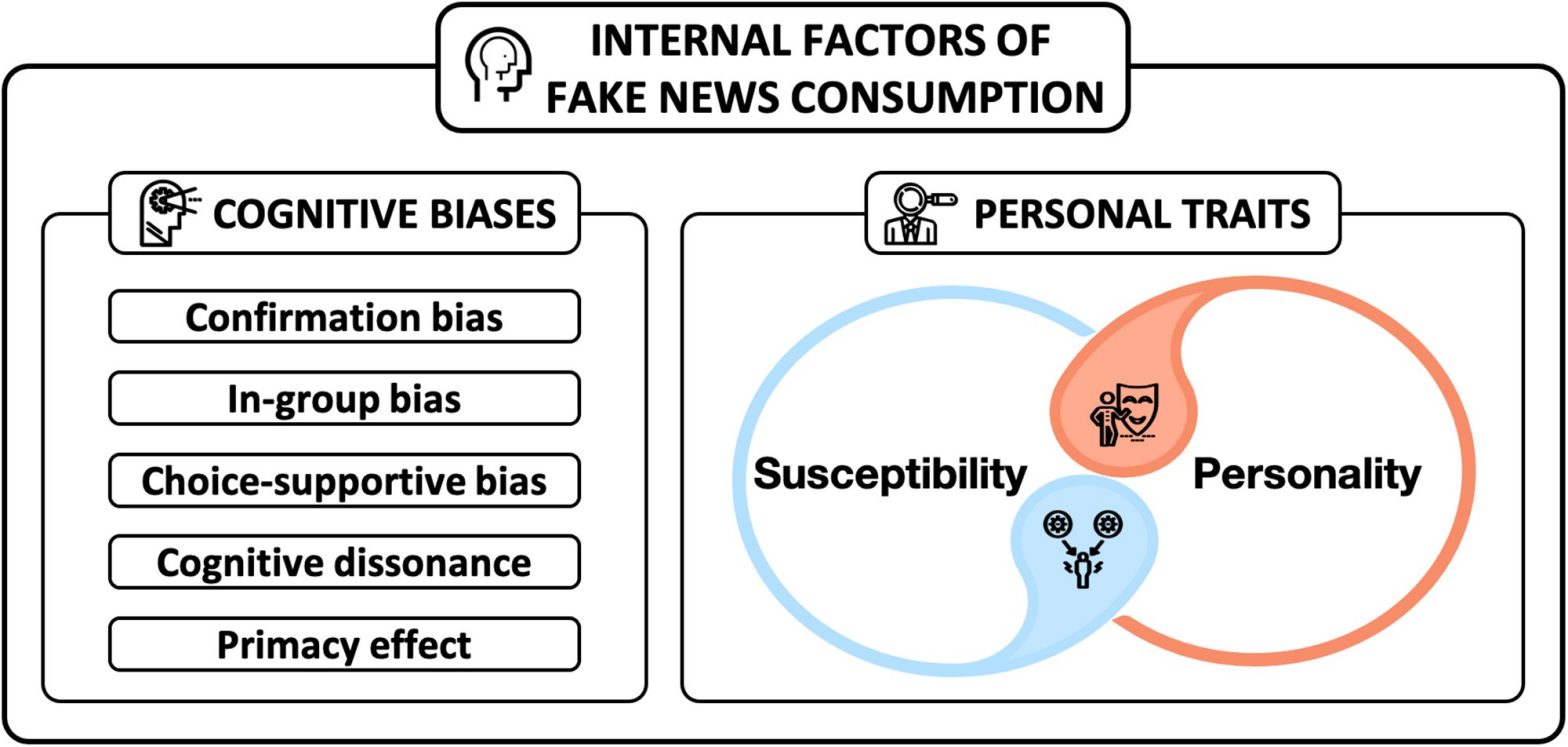
Two internal factors of fake news consumption: Cognitive biases and personal traits.

Due to the characteristics of the Internet and social media, people are accustomed to consuming information quickly, such as reading only news headlines and checking photos in news articles. This type of news consumption practice could lead people to consider news information mostly based on their beliefs or values. This practice can make it easier for people to fall into an echo chamber and further social confusion. We identified two internal factors affecting fake news consumption: *(1) cognitive biases and (2) personal traits* (see Fig 6).

#### 4.2.1 Cognitive biases

Cognitive bias is an observer effect that is broadly recognized in cognitive science and includes basic statistical and memory errors [8]. However, this bias may vary depending on what factors are most important to affect individual judgments and choices. We identified five cognitive biases that affect fake news consumption: confirmation bias, in-group bias, choice-supportive bias, cognitive dissonance, and primacy effect.

***Confirmation bias*** relates to a human tendency to seek out information in line with personal thoughts or beliefs, as well as to ignore information that goes against such beliefs. This stems from the human desire to be reaffirmed, rather than accept denials of one’s opinion or hypothesis. If the process of confirmation bias is repeated, a more solid belief is gradually formed, and the belief remains unchanged even after encountering logical and objective counterexamples. Evaluating information with an objective attitude is essential to properly investigating any social phenomenon. However, confirmation bias significantly hinders this. Kunda [122] discussed experiments that investigated the cognitive processes as a function of accuracy goals and directional goals. Her analysis demonstrated that people use different cognitive processes to achieve the two different goals. For those who pursue accuracy goals (reaching a “right conclusion”), information is used as a tool to determine whether they are right or not [123], and for those with directional goals (reaching a desirable conclusion), information is used as a tool to justify their claims. Thus, biased information processing is more frequently observed by people with directional goals [124].

People with directional goals have a desire to reach the conclusion they want. The more we emphasize the seriousness and omnipresence of fake news, the less people with directional goals can identify fake news. Moreover, their confirmation bias through social media could result in an echo chamber, triggering a differentiation of public opinion in the media. The algorithm of the media platform further strengthens the tendency of biased information consumption (e.g., filter bubble).

***In-group bias*** is a phenomenon in which an individual favors a group that he or she belongs to. The causes of in-group bias are two [125]. One is a categorization process, which exaggerates the similarities between members within one category (the internal group) and differences with others (the external groups). Consequently, positive reactions towards the internal group and negative reactions (e.g., hostility) towards the external group are both increased. The other reason is self-respect based on social identity theory. To positively evaluate the internal group, a member tends to perceive that other group members are similar to himself or herself.

In-group bias has a significant impact on fake news consumption because of radical changes in the media environment [126]. The public recognizes and forms groups based on issues through social media. The emotions and intentions of such groups of people online can be easily transferred or developed into offline activities, such as demonstrations and rallies. Information exchanges within such internal groups proceeds similarly to the situation with confirmation bias. If confirmation bias is keeping to one’s beliefs, in-group bias equates the beliefs of my group with my beliefs.

***Choice-supportive bias*** refers to an individual’s tendency to justify his or her decision by highlighting the evidence that he or she did not consider in making the decision [127]. For instance, people sometimes have no particular purpose when they purchase a certain brand of products or service, or support a particular politician or political party. They emphasize that their choices at the time were right and inevitable. They also tend to focus more on positive aspects than negative effects or consequences to justify their choice. However, these positive aspects can be distorted because they are mainly based on memory. Thus, choice-supportive bias, can be regarded as the cognitive errors caused by memory distortion.

The behavioral condition of choice-supportive bias is used to justify oneself, which usually occurs in the context of external factors (e.g., maintaining social status or relationships) [7]. For example, if people express a certain political opinion within a social group, people may seek information with which to justify the opinion and minimize its flaws. In this procedure, people may accept fake news as a supporting source for their opinions.

***Cognitive dissonance*** was based on the notion that some psychological tension would occur when an individual had two perceptions that were inconsistent [128]. Humans have a desire to identify and resolve the psychological tension that occurs when a cognitive dissonance is established. Regarding fake news consumption, people easily accept fake news if it is aligned with their beliefs or faith. However, if such news is seen as working against their beliefs or faith, people define even real news as fake and consume biased information in order to avoid cognitive dissonance. This is quite similar to cognitive bias. Selective exposure to biased information intensifies its extent and impact in social media. In these circumstances, an individual’s cognitive state is likely to be formed by information from unclear sources, which can be seen as a negative state of perception. In that case, information consumers selectively consume only information that can be in harmony with negative perceptions.

***Primacy effect*** means that information presented previously will have a stronger effect on the memory and decision-making than information presented later [129]. The “interference theory [130]” is often referred to as a theoretical basis for supporting the primacy effect, which highlights the fact that the impression formed by the information presented earlier influences subsequent judgments and the process of forming the next impression.

The significance of the primary effect for fake news consumption is that it can be a starting point for biased cognitive processes. If an individual first encounters an issue in fake news and does not go through a critical thinking process about that information, he or she may form false attitudes regarding the issue [131, 132]. Fake news is a complex combination of facts and fiction, making it difficult for information consumers to correctly judge whether the news is right or wrong. These cognitive biases induce the selective collection of information that feels more valid for news consumers, rather than information that is really valid.

#### 4.2.2 Personal traits

We two aspects of personal characteristics or traits can influence one’s behaviors in terms of news consumption: susceptibility and personality.

*4.2.2.1 Susceptibility*. The most prominent feature of social media is that consumers can be also creators, and the boundaries between the creators and consumers of information become unclear. New media literacy (i.e., the ability to critically and suitably consume messages in a variety of digital media channels, such as social media) can have a significant impact on the degree of consumption and dissemination of fake news [133, 134]. In other words, the higher new media literacy is, the higher the probability that an individual is likely to take a critical standpoint toward fake news. Also, the susceptibility level of fake news is related to one’s selective news consumption behaviors. Bessi et al. [35] studied misinformation on Facebook and found that users who frequently interact with alternative media tend to interact with intentionally false claims more often.

***Personality*** is an individual’s traits or behavior style. Many scholars have agreed that the personality can be largely divided into five categories (Big Five)—extraversion, agreeableness, neuroticism, openness, and conscientiousness [135, 136]—and used them to understand the relationship between personality and news consumption.

Extroversion is related to active information use. Previous studies have confirmed that extroverts tend to use social media and that their main purpose of use is to acquire information [137] and better determine the factuality of news on social media [138]. Furthermore, people with high agreeableness, which refers to how friendly, warm, and tactful, tend to trust real news than fake news [138]. Neuroticism refers to a broad personality trait dimension representing the degree to which a person experiences the world as distressing, threatening, and unsafe. People with high neuroticism usually show negative emotions or information sharing behavior [139]. Neuroticism is positively related to fake news consumption [138]. Openness refers to the degree of enjoying new experiences. High openness is associated with high curiosity and engagement in learning [140], which enhances critical thinking ability and decreases negative effects of fake news consumption [138, 141]. Conscientiousness refers to a person’s work ethic, being orderly, and thoroughness [142]. People with high conscientiousness tend to regard social media use as distraction from their tasks [143–145].

### 4.3 Fake news awareness and prevention

#### 4.3.1 Decision-making support tools

News on social media does not go through the verification process, because of its high degree of freedom to create, share, and access information. The study reported that most citizens in advanced countries will have more fake information than real information in 2022 [146]. This indicates that potential personal and social damage from fake news may increase. Paradoxically, many countries that suffer from fake news problems strongly guarantee the freedom of expression under their constitutions; thus, it would be very difficult to block all possible production and distribution of fake news sources through laws and regulations. In this respect, it would be necessary to put in place not only technical efforts to detect and prevent the production and dissemination of fake news but also social efforts to make news consumers aware of the characteristics of online fake information.

Inoculation theory highlights that human attitudes and beliefs can form psychological resistance by being properly exposed to arguments against belief in advance. To have the ability to strongly protest an argument, it is necessary to expose and refute the same sort of content with weak arguments first. Doris-Down et al. [147] asked people who were from different political backgrounds to communicate directly through mobile apps and investigated whether these methods alleviated their echo-chamberness. As a result, the participants made changes, such as realizing that they had a lot in common with people who had conflicting political backgrounds and that what they thought was different was actually trivial. Karduni et al. [148] provided comprehensive information (e.g., connections among news accounts and a summary of the location entities) to study participants through the developed visual analytic system and examined how they accepted fake news. Another study was conducted to confirm how people determine the veracity of news by establishing a system similar to social media and analyzing the eye tracking of the study participants while reading fake news articles [28].

Some research has applied the inoculation theory to gamification. A “Bad News” game was designed to proactively warn people and expose them to a certain amount of false information through interactions with the gamified system [29, 149]. The results confirmed the high effectiveness of inoculation through the game and highlighted the need to educate people about how to respond appropriately to misinformation through computer systems and games [29].

#### 4.3.2 Fake information propagation analysis

Fake information tends to show a certain pattern in terms of consumption and propagation, and many studies have attempted to identify the propagation patterns of fake information (e.g., the count of unique users, the depth of a network) [150–153].

*4.3.2.1 Psychological characteristics*. The theoretical foundation of research intended to examine the diffusion patterns of fake news lies in psychology [154, 155] because psychological theories explain why and how people react to fake news. For instance, a news consumer who comes across fake news will first have doubts, judge the news against his background knowledge, and want to clarify the sources in the news. This series of processes ends when sufficient evidence is collected. Then the news consumer ends in accepting, ignoring, or suspecting the news. The psychological elements that can be defined in this process are doubts, negatives, conjectures, and skepticism [156].

*4.3.2.2 Temporal characteristics*. Fake news exhibits different propagation patterns from real news. The propagation of real news tends to slowly decrease over time after a single peak in the public’s interest, whereas fake news does not have a fixed timing for peak consumption, and a number of peaks appear in many cases [157]. Tambuscio et al. [151] proved that the pattern of the spread of rumors is similar to the existing epidemic model [158]. Their empirical observations confirmed that the same fake news reappears periodically and infects news consumers. For example, rumors that include the malicious political message that “Obama is a Muslim” are still being spread a decade later [159]. This pattern of proliferation and consumption shows that fake news may be consumed for a certain purpose.

## 5 A mental-model approach

We have examined news consumers’ susceptibility to fake news due to internal and external factors, including personal traits, cognitive biases, and the contexts. Beyond an investigation on the factor level, we seek to understand people’s susceptibility to misinformation by considering people’s internal representations and external environments holistically [5]. Specifically, we propose to comprehend people’s *mental models* of fake news. In this section, we first briefly introduce mental models and discuss their connection to misinformation. Then, we discuss the potential contribution of using a mental-model approach to the field of misinformation.

### 5.1 Mental models

A mental model is an internal representation or simulation that people carry in their minds of how the world works [160, 161]. Typically, mental models are constructed in people’s working memory, in which information from long-term memory and the environments are combined [162]. They also indicate that individuals represent complex phenomena with somewhat abstraction based on their own experiences and understanding of the contexts. People rely on mental models to understand and predict their interactions with environments, artifacts and computing systems, as well as other individuals [163, 164]. Generally, individuals’ ability to represent the continually changing environments is limited and unique. Thus, mental models tend to be functional and dynamic but not necessarily accurate or complete [163, 165]. Mental models also differ between various groups and in particular between experts and novices [164, 166].

### 5.2 Mental models and misinformation

Mental models have been proposed to understand human behaviors in spatial navigation [167], learning [168, 169], deductive reasoning [170], mental presentations of real or imagined situations [171], risk communication [172], and usable cybersecurity and privacy [166, 173, 174]. People use mental models to facilitate their comprehension, judgment, and actions, and can be the basis of individual behaviors. In particular, the connection between a mental-model approach and misinformation has been revealed in risk communication regarding vaccines [175, 176]. For example, Downs et al. [176] interviewed 30 parents from three US cities to understand their mental models about vaccination for their children aged 18 to 23 months. The results revealed two mental models about vaccination: (1) *heath oriented*: parents who focused on health-oriented topics trusted anecdotal communication more than statistical arguments; and (2) *risk oriented*: parents with some knowledge about vaccine mechanisms trusted communication with statistical arguments more than anecdotal information. Also, the authors found that many parents, even those favorable to vaccination, can be confused by ongoing debate, suggesting somewhat incompleteness of their mental models.

### 5.3 Potential contributions of a mental-model approach

Recognizing and dealing with the plurality of news consumers’ perception, cognition and actions is currently considered as key aspects of misinformation research. Thus, a mental model approach could significantly improve our understanding of people’s susceptibility to misinformation, as well as inform the development of mechanisms to mitigate misinformation.

One possible direction is to investigate the demographic differences in the context of mental models. As more Americans have adopted social media, the social media users have become more representative for the population. Usage by older adults has increased in recent years, with the use rate of about 12% in 2012 to about 35% in 2016 (https://www.pewresearch.org/internet/fact-sheet/social-media/). Guess et al. (2019) analyzed participants’ profiles and their sharing activity on Facebook during the 2016 US Presidential campaign. A strong age effect was revealed. While controlled the effects of ideology and education, their results showed that Facebook users who are over 65 years old were associated with sharing nearly seven times as many articles from fake news domains on Facebook as those who are between 18–29 years old, or about 2.3 times as many as those in the age between 45 to 65.

Besides older adults, college students were shown more susceptibility to misinformation [177]. We can identify which mental models a particular age group ascribes to, and compare the incompleteness or incorrectness of the mental models by age. On the other hand, such comparison might be informative to design general mechanisms to mitigate misinformation independent of the different concrete mental models possessed by different types of users.

Users’ actions and decisions are directed by their mental models. We can also explore news consumers’ mental models and discover unanticipated and potentially risky human system interactions, which will inform the development and design of user interactions and education endeavors to mitigate misinformation.

A mental-model approach supplies an important, and as yet unconsidered, dimension to fake news research. To date, research on people’s susceptibility to fake news in social media has lagged behind research on computational aspect research on fake news. Scholars have not considered issues of news consumers’ susceptibility across the spectrum of their internal representations and external environments. An investigation from the mental model’s perspective is a step toward addressing such need.

## 6 Discussion and future work

In this section, we highlight the importance of balancing research efforts on fake news creation and consumption and discuss potential future directions of fake news research.

### 6.1 Leveraging insights of social science to model development

Developing fake news detection models has achieved great performance. Feature groups used in the model are diverse including linguistics, vision, sentiment, topic, user, and network, and many models used multiple groups to increase the performance. By using datasets with different size and characteristics, research has demonstrated the effectiveness of the models through a comparison analysis. However, much research has considered and used the features that are easily quantifiable, and many of them tend to have unclear justification or rationale of being used in modeling. For example, what is the relationship between the use of question (?), exclamation (!), or quotation marks (“…”) and fake news?, what does it mean by a longer description relates to news trustworthiness?. There are also many important aspects that can be used as additional features for modeling and have not yet found a way to be quantified. For example, journalistic styles are important characteristics that determine a level of information credibility [156], but it is challenging to accurately and reliably quantified them. There are many intentions (e.g., ideological standpoint, financial gain, panic creation) that authors may implicitly or explicitly display in the post but measuring them is uneasy and not straightforward. Social science research can play a role in here coming up with a valid research methodology to measure such subjective perceptions or notions considering various types and characteristics of them depending on a context or environment. Some research efforts in this research direction include quantifying salient factors of people’s decision-making identified in social science research and demonstrating the effectiveness of using the factors in improving model performance and interpreting model results [70]. Yet more research that applies socio-technical aspects in model development and application would be needed to better study complex characteristics of fake news.

#### 6.1.1 Future direction

Insights from social science may help develop transparent and applicable fake news detection models. Such socio-technical models may allow news consumers to have a better understanding of fake news detection results and its application as well as to take more appropriate actions to control fake news phenomenon.

### 6.2 Lack of research on fake news consumption

Regarding fake news consumption, we confirmed that only few studies involve the development of web- or mobile-based technology systems to help consumers aware possible dangers of fake news. Those studies [28, 29, 147, 148] tried to demonstrate the feasibility of developed self-awareness systems through user studies. However, due to the limited number of study participants (min: 11, max: 60) and their lack of demographic diversity (i.e., recruited only college students of one school, the psychology research pool at the authors’ institution), the generalization and applicability of these systems are still questionable. On the other hand, research that involves the development of fake news detection models or network analysis to identify the pattern of fake news propagation has been relatively active. These results can be used to identify people (or entities) who intentionally create malicious fake content; however, it is still challenging to restrict people who originally had not shown any behaviors or indications of sharing or creating fake information but later manipulated real news to fake or disseminated fake news with their malicious intention or cognitive biases.

In other words, although fake news detection models have shown great, promising performance, the influence of the models may be exerted in limited cases. This is because fake news detection models heavily rely on the data that were labeled as fake by other fact-checking institutions or sites. If someone manipulates the news that were not covered by fact-checking, the format or characteristics of the manipulated news may be different from those (i.e., conventional features) that are identified and managed in the detection model. Such differences may not be captured by the model. Therefore, to prevent fake news phenomenon more effectively, research needs to consider changes of news consumption.

#### 6.2.1 Future direction

It may be desirable to support people recognizing that their news consumption behaviors (e.g., like, comment, share) can have a significant ripple effect. Developing a system that tracks activities of people’s news consumption and creation, measures similarity and differences between those activities, and presents behaviors or patterns of news consumption and creation to people would be helpful.

### 6.3 Limited coverage of fact-checking websites and regulatory approach

Some of the well-known fact-checking websites (e.g., snopes.com, politifact.com) cover news shared mostly on the Internet and label the authenticity or deficiencies of the content (e.g., miscaptioned, legend, misattributed). However, these fact-checking websites may show limited coverage in that they are only used for those who are willing to check the veracity of certain news articles. Social media platforms have been making continuous efforts to mitigate the spread of fake news. For example, Facebook shows that content that has been falsely assessed by fact-checkers is relatively less exposed to news feeds or shows warning indicators [178]. Instagram has also changed the way that warning labels are displayed when users attempt to view the content that has been falsely assessed [179]. However, this type of an interface could lead news consumers to relying on algorithmic decision-making rather than self-judgment because these ostensible regulations (e.g., warning labels) tend to lack transparency of the decision. As we explained previously, this is related to filter bubbles. Therefore, it is important to provide a more clear and transparent communicative interface for news consumers to access and understand underlying information of the algorithm results.

#### 6.3.1 Future direction

It is necessary to create a news consumption circumstance that gives a wider coverage of fake news and more transparent information of algorithmic decisions on news credibility. This will help news consumers preemptively avoid fake news consumption and contribute more to preventing fake news propagation. Consumers also make more proper and accurate decisions based on their understanding of the news.

### 6.4 New media literacy

With the diversification of news channels, we can easily consume news. However, we are also in a media environment that asks us to self-critically verify news content (e.g., whether the news title reads like a clickbait, whether the news title and content are related), which in reality is hard to be done. Moreover, in social media, news consumers can be news creators or reproducers. During this process, news information could be changed based on a consumer’s beliefs or interests. A problem here is that people may not know how to verify news content or not be aware of whether the information could be distorted or biased. As the news consumer environment changes rapidly and faces modern media deluge, the importance of media literacy education is high. Media literacy refers to the ability to decipher media content, but in a broad sense, to understand the principles of media operation and media content sensibly and critically, and in turn to the ability to utilize and creatively reproduce content. Being a “lazy thinker” is more susceptible to fake news than having a “partisan bias” [32]. As “screen time” (i.e., time spent looking at smartphone, computer, or television screens) has become more common, people are consuming only stimulating (e.g., sensual pleasure and excitement) information [180]. This could gradually lower one’s ability of critical, reasonable thinking, leading to making wrong judgments and actions. In France, when fake news problem became more serious, and a great amount of efforts were made to create “European Media Literacy Week” in schools [181]. The US is also making legislative efforts to add media literacy to the general education curriculum [182]. However, the acquisition of new media literacy through education may be limited to people in school (e.g., young students) and would be challenging to be expanded to wider populations. Thus, there is also a need for supplementary tools and research efforts to support more people to critically interpret and appropriately consume news.

In addition, more critical social attention is needed because visual content (e.g., images, videos), which had been naturally accepted as facts, can be easily manipulated in a malicious fashion and looked very natural. We have seen that people prefer to watch YouTube videos for news consumption rather than reading news articles. This visual content makes it relatively easy for news consumers to trust the content compared to text-based information and makes it easier to obtain information simply by playing the video. Since visual content will become a more dominant medium in future news consumption, educating and inoculating news consumers about potential threats of fake information in such news media would be important. More attention and research are needed on the technology supporting fake visual content awareness.

#### 6.4.1 Future direction

Research in both computer science and social science should find ways (e.g., developing a game-based education system or curriculum) to help news consumers aware of their practice of news consumption and maintain right news consumption behaviors.

## 7 Conclusion

We presented a comprehensive summary of fake news research through the lenses of news creation and consumption. The trends analysis indicated a growing increase in fake news research and a great amount of research focus on news creation compared to news consumption. By looking into internal and external factors, we unpacked the characteristics of fake news creation and consumption and presented the use of people’s mental models to better understand people’s susceptibility to misinformation. Based on the reviews, we suggested four future directions on fake news research—(1) a socio-technical model development using insights from social science, (2) in-depth understanding of news consumption behaviors, (3) preemptive decision-making and action support, and (4) educational, new media literacy support—as ways to reduce the gap between news creation and consumption and between computer science and social science research and to support healthy news environments.

## Supporting information

S1 Checklist (PDF)
