# Correction: A systematic review on fake news research through the lens of news creation and consumption: Research efforts, challenges, and future directions

**DOI:** 10.1371/journal.pone.0296554

**Published:** 2023-12-28

**Authors:** The PLOS One Staff

There are errors in the Funding section. The correct Funding statement is: This research was supported by the Institute of Information & communications Technology Planning & Evaluation (IITP) grant funded by the Korea government (2019-0-01584, 2020-0-01373, IITP-2021-2018-0-01431).

The publisher apologizes for the error.
